# Integrative analysis of the metabolome and transcriptome reveals the molecular mechanism of chlorogenic acid synthesis in peach fruit

**DOI:** 10.3389/fnut.2022.961626

**Published:** 2022-07-19

**Authors:** Ziwen Su, Haoran Jia, Meng Sun, Zhixiang Cai, Zhijun Shen, Bintao Zhao, Jiyao Li, Ruijuan Ma, Mingliang Yu, Juan Yan

**Affiliations:** ^1^Institute of Pomology, Jiangsu Academy of Agricultural Sciences, Jiangsu Key Laboratory for Horticultural Crop Genetic Improvement, Nanjing, China; ^2^College of Horticulture, Nanjing Agricultural University, Nanjing, China; ^3^College of Agriculture and Biotechnology, Zhejiang University, Hangzhou, China

**Keywords:** peach, chlorogenic acid, metabolome, transcriptome, candidate genes

## Abstract

As the most abundant phenolic acid in peach fruit, chlorogenic acid (CGA) is an important entry point for the development of natural dietary supplements and functional foods. However, the metabolic and regulation mechanisms underlying its accumulation in peach fruits remain unclear. In this study, we evaluated the composition and content of CGAs in mature fruits of 205 peach cultivars. In peach fruits, three forms of CGA (52.57%), neochlorogenic acid (NCGA, 47.13%), and cryptochlorogenic acid (CCGA, 0.30%) were identified. During the growth and development of peach fruits, the content of CGAs generally showed a trend of rising first and then decreasing. Notably, the contents of quinic acid, shikimic acid, p-coumaroyl quinic acid, and caffeoyl shikimic acid all showed similar dynamic patterns to that of CGA, which might provide the precursor material basis for the accumulation of CGA in the later stage. Moreover, CGA, lignin, and anthocyanins might have a certain correlation and these compounds work together to maintain a dynamic balance. By the comparative transcriptome analysis, 8 structural genes (*Pp4CL*, *PpCYP98A*, and *PpHCT*) and 15 regulatory genes (*PpMYB*, *PpWRKY*, *PpERF*, *PpbHLH*, and *PpWD40*) were initially screened as candidate genes of CGA biosynthesis. Our findings preliminarily analyzed the metabolic and molecular regulation mechanisms of CGA biosynthesis in peach fruit, which provided a theoretical basis for developing high-CGA content peaches in future breeding programs.

## Introduction

Chlorogenic acid (CGA) is a phenolic compound produced from caffeic acid (CA) and quinic acid (QA) and an important secondary metabolite in the phenylpropanoid pathway (1). CGA is widely distributed in plants from dicots to ferns. In honeysuckle, coffee, chrysanthemum, and tobacco, the CGA contents are very high (2, 3). Potato, tomato, carrot, spinach, and other vegetables also contain CGA (4). In addition, CGA is the main phenolic in many fruits, such as peach, blueberry, pear, strawberry, and apple (5–8). CGA has multiple biological activities, including free radical scavenging and the reduction of blood pressure and blood lipid content, along with anti-bacterial and anti-inflammatory properties (9). Thus, CGA has become a hot topic in active substance research and is used widely in aspects of the food, cosmetics, medicinal, and chemical industries.

CGA is synthesized *via* the phenylpropanoid pathway, and the biosynthetic routes of CGA in plants are not yet completely defined. To date, three CGA biosynthetic pathways have been proposed. In route 1, hydroxycinnamoyl-CoA quinate hydroxycinnamoyl transferase (HQT, EC 2.3.1.99) catalyzes the formation of CGA from caffeoyl-CoA and quinic acid (2), as in tobacco and tomato. This route is the most common pathway for CGA biosynthesis. In route 2, caffeoyl-CoA is supplied by the combined activities of hydroxycinnamoyl-CoA shikimate/quinate hydroxycinnamoyl transferase (HCT, EC 2.3.1.133) and p-coumaroyl ester 3′-hydroxylase (C3′H, EC 1.14.13.36) *via* a p-coumaroylshikimate intermediate (10). HCT and C3′H are both active in *Arabidopsis thaliana*, but no accumulation of CGA occurs, indicating that this is not the main synthesis pathway (11). In route 3, CGA is produced from coumaroyl quinic acid and caffeoyl-D-glucose by the mediation of hydroxycinnamoyl D-glucose: quinate hydroxycinnamoyl transferase (HCGQT), as in sweet potato (12).

The molecular mechanism underlying CGA accumulation has been studied extensively in numerous plants. Many structural genes are involved in CGA biosynthesis by encoding enzymes, such as phenylalanine ammonia lyase (PAL), 4-coumarate coenzyme A ligase (4CL), cinnamate 4-hydroxylase (C4H), coumarate 3-hydroxylase (C3H), hydroxycinnamoyl transferase (HCT), and HQT. As crucial rate-limiting enzymes involved in the first three steps of CGA biosynthesis, the specific details of the roles of PAL, C4H, and 4CL in this process have not yet been fully elucidated (3). In sweet potato, overexpression of *IbPAL1* promotes CGA accumulation and the expression of genes involved in the biosynthetic pathway in leaves, stimulates secondary xylem cell expansion in stems and inhibits storage root formation (13). In *Lonicera japonica*, the CGA levels in the leaves are positively correlated with *LjC3H* transcript abundance (14). Another study reported that *LjC4H2*, encoding C4H, is critical in regulating CGA biosynthesis (15). As key enzymes involved downstream of CGA biosynthesis, the rate-limiting role of HCT and HQT has been demonstrated in many plants (10). In *Cynara cardunculus*, the *HQT* gene is involved in the synthesis of CGA (3, 16). In *Populus trichocarpa*, the expression of *PtHCT2* in leaf tissues is significantly correlated with CGA abundance (17). And in *Taraxacum antungense*, the concentrations of 5-caffeoylquinic acid in calli were found to be reduced by *TaHQT1* and *TaHQT2* knockdown, while the inoculation of *T. antungense* plants tissues with recombinant *TaHQT1* and *TaHQT2* increased 5-caffeoylquinic acid levels *in situ* (18).

Some transcription factors (TFs) are also reported to regulate the biosynthesis of CGA. In suspension-cultured carrot cells, *DcMYB3* and *DcMYB5* activated the transcription of *DcPAL3*, resulting in a high level of CGA accumulation (19). Similarly, the biosynthesis of many phenolic compounds has been recorded to be regulated by the WRKY family. For example, five *PtWRKYs* (38, 45, 60, 89, and 93) were shown to act as activators for *PtHCT2* in poplar. When the five *PtWRKYs* were transiently overexpressed in poplar protoplasts, the expression level of *PtHCT2* was significantly increased (17). In *T. antungense*, *TaWRKY14* binds to the W-box of *proTaPAL1*, and its overexpression enhances the accumulation of CGA and increases the resistance to powdery mildew (20). Moreover, in *L. japonica*, the TF *LjbZIP8* binds specifically to the G-box element of the *LjPAL2* 5′-UTR and regulates the synthesis of CGA by inhibiting the expression of *PALs* (21). In *T. antungense*, *TabHLH1* binds directly to the bHLH-binding motifs of proTaHQT2 and proTa4CL, and the concentrations of CGA and luteolin are significantly higher in the *OE-TabHLH1* transgenic line (22). In *Salvia miltiorrhiza*, the overexpression of *ERF115* increases phenolic acid content, but silencing it reduces the level of this acid (23). In *Platycodon grandiflorum*, the over-expression of an *A. thaliana* TF *AtPAP1* leads to the enhancement of CGA production in hairy roots (24).

Peach [*Prunus persica* (L.) Batsch] is native to China and popular among consumers because of its beautiful appearance, delicate flesh aromatic flavor, and high nutritional value. As the phenolic acid with the highest content in peach fruit, CGA is a beneficial ingredient with multiple functions (9). This is an important entry point for the development of natural dietary supplements and functional foods. However, studies on CGA in peach have focused mainly on content determination and extraction methods, whereas there are few studies on the regulatory mechanism and gene function associated with CGA biosynthesis (25, 26). In this study, we evaluated the content and composition of CGA compounds in the ripe fruits of 205 peach cultivars. Moreover, two high- and two low-CGAs cultivars were selected to investigate the dynamic changes of various metabolites involved in CGA biosynthesis pathways throughout fruit development. Furthermore, several candidate structural genes and TFs for CGA accumulation were identified using the comparative transcriptome analysis strategy. This study aims to provide insight into the metabolic and molecular regulation mechanisms of CGA accumulation in peach fruit, which will be helpful in future breeding programs aimed at quality and resistance breeding.

## Materials and methods

### Plant materials

The experiment was conducted at the National Peach Fruit Germplasm Repository of Nanjing, China, from April to August 2020. In this experiment, 205 peach germplasm resources were used as the experimental materials (Supplementary Table 1). There were 153 common peach cultivars, 29 nectarine peach cultivars, and 22 flat peach cultivars, including 134 white flesh peaches, 52 yellow flesh peaches, and 19 blood flesh peaches. Mature fruits were randomly collected according to fruit skin background color, size, and solidity indicators. Furthermore, two cultivars with a high content of total CGAs (‘BJ’ and ‘HYT’) and two cultivars with a low content of total CGAs (‘XH’ and ‘RG’) were selected to investigate the dynamic accumulation patterns of CGAs throughout fruit development. According to the growth and development curve for peach fruit, five time points, young fruit stage (S1), pit hardening (S2), expansion stage (S3), pre-mature stage (S4_1), and mature stage (S4_2), were selected for specific analysis. For each cultivar, fruits from ‘BJ’ were selected at 40, 50, 70, 80, and 90 days after flowering (DAF), ‘XH’ at 40, 60, 80, 90, and 96 DAF, ‘RG’ at 40, 80, 100, 110, and 120 DAF, ‘HYT’ at 40, 80, 100, 120, and 128 DAF. Each cultivar contained three biological replicates, and each biological replicate contained at least five fruits. All of the fruit samples were separated into skin and flesh, and then immediately frozen in liquid nitrogen and stored at −80°C. Part of the samples was used for RNA extraction, and the other part was used for metabolite determination.

### Measurement of various metabolites involved in the chlorogenic acid synthesis pathways of peach

The extraction of organic acids from peach fruits was performed according to the method described by Zheng et al. (27). First, 1.0 g of peach fruit ground sample and 5 mL of deionized water were placed in a centrifuge tube. Then, the mixed solution was ultrasound extracted for 30 min and centrifuged for 10 min. Finally, the supernatant was filtered through a 0.22 μm water filter and added to the chromatographic column. The organic acid contents were measured using High Performance Liquid Chromatography (Agilent 1260, CA, United States). Chromatographic separation was conducted using an Agilent ZORBAX SB-Aq (4.6 mm × 250 mm, 5 μm), with 0.2% HPO_3_ solution as the mobile phase. The elution was performed at a flow rate of 0.5 mL/min and the column temperature was maintained at 25°C. The contents of organic acids (quinic acid and shikimic acid) were quantified using a diode array detector (DAD), with absorbance detected at 217 nm.

The extraction of phenolic substances from peach fruits was performed according to the method described by Yan et al. (25). First, 1.0 g of peach fruit ground sample and 5 mL of methanol (0.1% H_3_PO_4_) extractant were added to a centrifuge tube. Then, the mixed solution was ultrasound extracted for 30 min in the dark and centrifuged at 4°C for 10 min. Finally, the supernatant was filtered through a 0.22 μm organic filter and added to a chromatographic column.

The phenolic acid contents were also measured using High Performance Liquid Chromatography (Agilent 1260, CA, United States). Chromatographic separation was conducted with an Agilent ZORBAX SB-C18 column (4.6 mm × 250 mm, 5 μm) and a binary solvent system of (A) methanol (0.1% H_3_PO_4_) and (B) water (0.1% H_3_PO_4_). The elution was performed at a flow rate of 1.0 mL/min and the column temperature was maintained at 30°C. The contents of phenolic acids [CGA, neochlorogenic acid (NCGA), cryptochlorogenic acid (CCGA), p-coumaryl quinic acid, and caffeoyl shikimic acid] were detected at 320 nm and the contents of anthocyanin (cyanidin-3-O-glucoside chloride and cyanidin 3-O-rutinoside chloride) were quantified at 525 nm. All standards (≥ 98%) were purchased from Solarbio (Solarbio, Beijing, China). The contents of metabolites were analyzed in triplicate and calculated based on peak area measurements.

Lignin was extracted from peach fruit using a micro-lignin detection kit (Solarbio, Beijing, China). First, the samples were dried, crushed, and sieved. Then, 3.0 mg of the samples were acetylated for 40 min at 80°C. Finally, the absorbance of the supernatant was measured at 280 nm. Each sample consisted of three biological replicates.

### RNA extraction, library construction, and sequencing

Transcriptomic sequencing was completed by Beijing Nuohe Zhiyuan Technology Co., Ltd. Total RNA was extracted from the fruit samples, and the amounts and integrity of RNA were assessed using Agilent 2100 bioanalyzer. Using poly-T oligo-attached magnetic beads, mRNA was purified from total RNA. Using the short fragmented mRNA as the template, the first-strand cDNA was synthesized, and then the second cDNA strand was synthesized. The purified double-stranded cDNAs were end-repaired, added A-tailed, and ligated with sequencing adaptors. Subsequently, the cDNA fragments of preferentially 370–420 bp in length were selected and PCR amplified, and the library was finally obtained. After the library was qualified, the different libraries were pooling according to the effective concentration and the target amount of data off the machine, then being sequenced by the Illumina NovaSeq 6000. The end reading of 150 bp pairing was generated. The basic principle of sequencing was to synthesize and sequence at the same time (Sequencing by Synthesis).

### Transcriptome data analysis

The image data measured by the high-throughput sequencer were converted into sequence data (reads) by CASAVA base recognition. Clean reads were obtained by removing reads containing adapter, reads containing Nbase, and low-quality reads. Concurrently, the Q20, Q30, and GC content were calculated. The clean reads were mapped to the reference genome (GCF_000346465.2) of peach using HISAT2. Estimation of gene expression levels was performed based on the value of the expected number of fragments per kilobase of transcript sequence per million base pairs sequenced (FPKM). Identification of differentially expressed genes (DEGs) was conducted using the program DESeq2. The resulting *P*-values were adjusted for controlling the false discovery rate. “padj ≤ 0.05 and log_2_(foldchange) ≥ 1” were set as the threshold for significantly differential expression.

### Pathway enrichment analysis

To obtain more detailed information about DEGs, we performed enrichment analysis using Gene Ontology (GO^1^) and Kyoto Encyclopedia of Genes and Genomes (KEGG^2^) databases. All DEGs mapped in GO and KEGG pathways with the *P*-value of ≤0.05 were considered significantly enriched. GO is an international website for functional annotations of genes, providing a range of semantics for describing the characteristics of genes and gene products. KEGG is a database resource for understanding high-level functions and utilities of the biological system, such as the cell, the organism, and the ecosystem, from molecular-level information, especially large-scale molecular datasets generated by genome sequencing and other high-throughput experimental technologies.

### Weighted correlation network analysis

We identified gene coexpression modules related to CGA biosynthesis by weighted correlation network analysis (WGCNA). WGCNA is a systematic biological method used to describe the gene association modes among different samples. It can be used to identify gene sets that are highly synergistic changed, and identify candidate biomarkers or therapeutic targets based on the coherence of gene sets and the correlation between gene sets and phenotypes. The R package WGCNA is a set of functions used to calculate various weighted association analysis, which can be used for network construction, gene screening, gene cluster identification, topological feature calculation, data simulation, and visualization (28). WGCNA is suitable for multisample data. Generally, more than 15 samples are required. One input file is sample information, that is, a matrix describing the traits of the sample: the traits used for association analysis must be numeric; if it is a regional or categorical variable, it needs to be converted to a 0–1 matrix. The other is gene expression data. For transcriptome sequencing, FPKM can be used as gene expression data.

### Validation of RNA-Seq by quantitative real-time polymerase chain reaction

To validate the accuracy of the gene expression levels of DEGs obtained from the RNA-Seq analysis, 12 DEGs associated with CGA synthesis were randomly selected and subjected to quantitative real-time polymerase chain reaction (qRT-PCR) detection. According to the design principle of qRT-PCR primer, the gene-specific primers were designed on Primer 5.0 (Supplementary Table 2). The cDNA synthesis was performed using the Hifair^®^ II 1st Strand cDNA Synthesis SuperMix for qPCR (Yeasen, Shanghai, China). qRT-PCR was conducted following the instructions of Hieff^®^ qPCR SYBR^®^ Green Master Mix (Yeasen, Shanghai, China). The amplification system was 20 μL: cDNA 1 μL, upstream and downstream primer 0.8 μL each, Hieff^®^ qPCR SYBR^®^ Green Master Mix 10 μL, ddH_2_O 7.4 μL. qRT-PCR was performed using the Applied Biosystems 7500 Real-Time PCR System (Applied Biosystems, United States), with the following program: 95°C for 15 s, 60°C for 35 s, 40 cycles. All gene expression tests were performed with three biological replicates and three technical replicates. The relative expression level was calculated with the formula 2^–ΔΔCT^ = normalized expression ratio.

## Results

### Identification of the composition and content of chlorogenic acids in the peach germplasm resources

**FIGURE 1:**
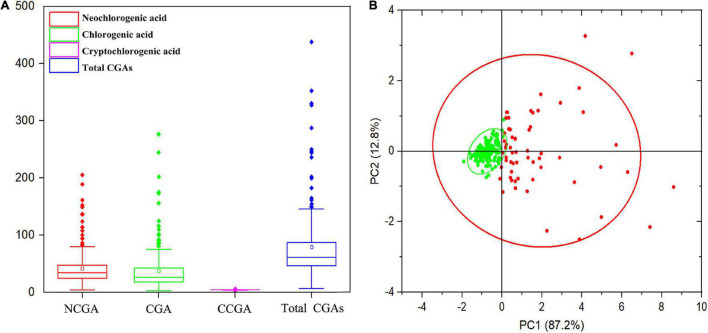
Range and distribution of CGAs contents in ripe fruits of 205 peach accessions. **(A)** The box plot of the CGAs contents distribution. **(B)** Principal component analysis (PCA) of CGAs contents. The variance explained by each component (%) was given in parentheses. All tested accessions were divided into two separate groups that were highlighted in different colors. Green dots represented accessions with lower total CGAs than average, whereas, red dots represented accessions with higher total CGAs than average.

In this study, the content and composition of CGAs in the mature fruits of 205 peach cultivars were measured, and the results are shown in Supplementary Table 1. A total of three CGA compounds were identified, including NCGA, CGA, and CCGA, with peak times of 9.746, 12.286, and 12.735 min (Supplementary Figure 1) and average concentrations of 40.79, 45.50, and 0.26 mg/kg FW, respectively (Figure 1A). CGA and NCGA were the major CGAs, with ranges of 3.12–306.19 and 4.12–205.63 mg/kg FW, which accounted for 52.57 and 47.13% of the total CGAs, respectively. However, the CCGA concentration was extremely low, with a range of 0.02–2.70 mg/kg FW, which only accounted for 0.30% of the total CGAs. In addition, total CGAs content (the sum of CGA, NCGA, and CCGA) ranged from 7.50 to 458.92 mg/kg FW, with an average of 86.55 mg/kg FW. In all accessions, the content of total CGAs showed a normal distribution with a skew to the left, 144 (70.24%) varieties had lower levels than average, while 61 (29.76%) varieties had higher levels than average (Figure 1B). Among the tested accessions, ‘HJM’ had the lowest contents of NCGA (4.12 mg/kg FW) and CGA (3.12 mg/kg FW); ‘HYH’ had the highest content of NCGA (205.63 mg/kg FW); and ‘YTSMT’ and ‘BJYXH’ had the highest contents of CGA, which were 304.29 and 306.19 mg/kg FW, respectively.

The CGA compounds of peaches with different flesh-color were compared (Supplementary Figure 2A). In the white flesh and red flesh peach germplasm resources, the contents of CGA > NCGA > CCGA; while in the yellow flesh peach germplasm resources, the contents of NCGA > CGA > CCGA. The contents of total CGAs in red flesh peaches (155.88 mg/kg FW) were significantly higher than in yellow flesh (89.77 mg/kg FW) and white flesh peaches (75.47 mg/kg FW), with no significant difference between fruits with yellow or white flesh. In addition, the CGA compounds of peaches with different fruit types peaches were compared (Supplementary Figure 2B). In common peach germplasm resources, the contents of CGA > NCGA > CCGA; while in flat peach and nectarine germplasm resources, the contents of NCGA > CGA > CCGA. The content of total CGAs in common peach (91.19 mg/kg FW) > flat peach (74.06 mg/kg FW) > nectarine (71.39 mg/kg FW); the CGA content of common peach (50.10 mg/kg FW) > flat peach (35.28 mg/kg FW) > nectarine peach (28.87 mg/kg FW); the NCGA content of nectarine peach (42.24 mg/kg FW) > common peach (40.84 mg/kg FW) > flat peach (38.52 mg/kg FW); and the CCGA content of nectarine peach (0.28 mg/kg FW) > flat peach (0.26 mg/kg FW) and common peach (0.26 mg/kg FW). In summary, in terms of total CGAs and CGA, the contents in common peach were highest, followed by flat peach and nectarine peach; in terms of NCGA and CCGA, the contents in nectarine peach were highest, but there was little difference in different fruit types peaches.

### Dynamic changes of metabolites involved in the chlorogenic acid synthesis pathways during peach fruit development

**FIGURE 2:**
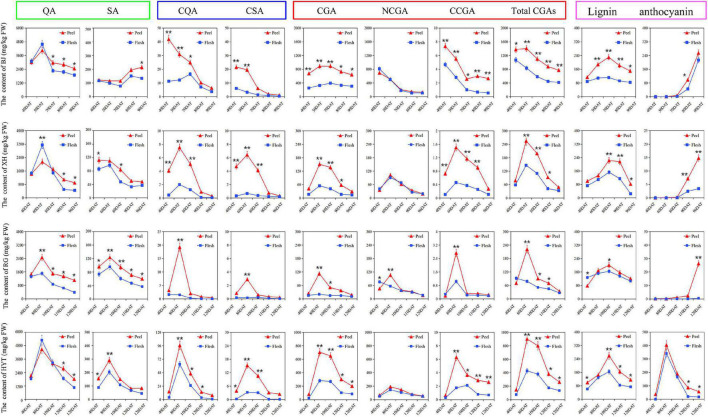
The dynamic change of various metabolites on the CGA synthesis pathways. Five time points were selected for specific analysis, fruits of ‘BJ’ were selected at 40, 50, 70, 80, and 90 DAF, ‘XH’ at 40, 60, 80, 90, and 96 DAF, ‘RG’ at 40, 80, 100, 110, and 120 DAF, ‘HYT’ at 40, 80, 100, 120, and 128 DAF. Green box, blue box, red box, and purple box represented the participating metabolome (quinic acid, QA; shikimic acid, SA), intermediate metabolites (p-coumaroyl quinic acid, PQA; caffeoyl shikimic acid, CSA), target metabolites (chlorogenic acid, CGA; neochlorogenic acid, NCGA; cryptochlorogenic acid, CCGA; total chlorogenic acids, total CGAs) and downstream metabolites (lignin and anthocyanin), respectively. Asterisks indicate a significant difference between peel and flesh at each time point as determined by Student’s *t*-test (*P < 0.05; **P < 0.01).

Based on the above PCA results, two cultivars containing high levels of total CGAs (‘BJ’ and ‘HYT’) and two cultivars containing low levels of total CGAs (‘XH’ and ‘RG’) were selected to investigate the accumulation rules of various metabolites on the CGA synthesis pathways during peach fruit development. Those tested included two participating metabolomes (quinic acid and shikimic acid), two intermediate metabolites (p-coumaryl quinic acid and caffeoyl shikimic acid), three target metabolites (CGA, NCGA, and CCGA), and two downstream metabolites (lignin and anthocyanin) (Figure 2). Among the four cultivars studied, the contents of total CGAs in ‘BJ’ were the highest, followed by ‘HYT’, while the contents in ‘XH’ and ‘RG’ were the lowest. In addition, the contents of total CGAs in peel were significantly higher than those in the flesh. At the mature fruit stage (S4), the contents of total CGAs in the peel were 772.64, 260.39, 48.52, and 34.05 mg/kg FW, and the contents of total CGAs in the flesh were 416.18, 134.58, 32.87, and 27.19 mg/kg FW, respectively. Of note, the trends in the contents of total CGAs in the four peach fruit cultivars were relatively consistent. During the growth and development of peach fruits, the content of total CGAs generally showed a trend of rising first and then decreasing. All four peach cultivars reached their peak of CGAs accumulation at the pit hardening stage (S2). However, due to the different growth periods of the four peach cultivars, the timings of these peaks differed. In general, ‘BJ’ exhibited their highest levels of total CGAs at 50 DAF, ‘XH’ at 60 DAF, ‘RG’ and ‘HYT’ at 80 DAF.

Furthermore, as important organic acids in the peach fruit, quinic acid and shikimic acid were two participating metabolomes of the CGA synthesis pathways, and they showed similar dynamic patterns to that of the total CGAs. The content of quinic acid was significantly higher than that of shikimic acid in all four peach cultivars, indicating that quinic acid might be the main metabolite involved in the CGA pathways. Similarly, as two important intermediate metabolites of the CGA metabolism pathway, p-coumaryl quinic acid and caffeoyl shikimic acid also showed similar dynamic patterns to that of the total CGAs, and their accumulation peaks were slightly earlier than that of the CGAs, indicating that they provided the precursor material for the accumulation of CGAs in the later stage of fruit development. Moreover, as a downstream metabolite of the phenylpropane metabolic pathway, lignin also showed a similar dynamic pattern to that of the CGAs, and its accumulation peak occurred later. Among these four cultivars, ‘BJ’ had the highest lignin content at 70 DAF, ‘XH’ at 80 DAF, ‘RG’ and ‘HYT’ at 100 DAF. However, as another downstream metabolite of the phenylpropane metabolism pathway, anthocyanin showed different trends from that of the CGAs. In ‘HYT’, anthocyanin showed a trend of rising at first and then falling; while in ‘BJ’, ‘XH’, and ‘RG’, it showed a rapid upward trend during the later fruit development stage.

### Identification of differentially expressed genes by comparative transcriptome analysis

**FIGURE 3:**
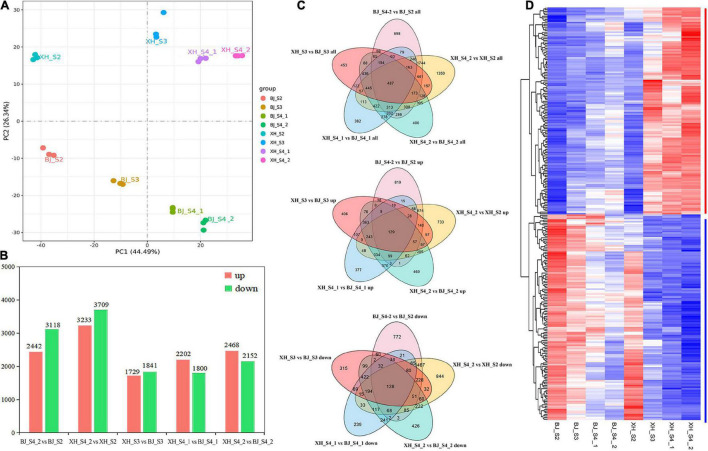
Identification of the DEGs. **(A)** Principal component analysis of samples. **(B)** Statistics of the number of DEGs. **(C)** Venn diagram of DEGs. **(D)** Cluster heatmap analysis of DEGs.

To investigate the potential molecular mechanisms of CGA biosynthesis in peach, samples of ‘BJ’ (high-CGAs cultivar) and ‘XH’ (low-CGAs cultivar) were selected at four later developmental stages for comparative dynamic transcriptome analysis, and each sample consisted of three independent replicates (Figure 3A). After read filtering, a total of 154.26 Gb clean reads were obtained from the 24 samples, with an average of 6.43 Gb clean reads per sample, of which 92.10–95.69% clean reads were uniquely mapped to the peach reference genome. The Q20 percentages ranged from 97.94 to 98.16%, while the Q30 percentages ranged from 93.39 to 94.60% (Supplementary Table 3). These data showed that the RNA-Seq was of high quality, and the data could be used for further analysis. Furthermore, based on the CGA accumulation patterns during peach fruit development, five comparison groups (BJ_S4_2 vs. BJ_S2, XH_S4_2 vs. XH_S2, XH_S3 vs. BJ_S3, XH_S4_1 vs. BJ_S4_1, XH_S4_2 vs. BJ_S4_2) were selected, which produced 5560, 6942, 3570, 4002, and 4620 DEGs, respectively (Supplementary Table 4 and Figure 3B). By analyzing the five different comparison groups, a total of 487 DEGs were obtained. Among them, 128 candidate genes (down-regulated genes) were positively correlated with the accumulation pattern of CGAs, and 129 candidate genes (up-regulated genes) were negatively correlated with the accumulation pattern of CGAs, which were further screened as candidate genes (Figures 3C,D).

To determine the biological function of DEGs, we annotated DEGs into the GO database, which included cellular component (CC), molecular function (MF), and biological process (BP) three parts (Supplementary Figure 3A). A total of 128 down-regulated candidate genes were annotated into 391 GO pathways, including 258 BP (65.98%), 44 CC (11.25%), and 89 MF (22.76%). The main enrich pathways included pigment metabolic process (GO:0042440), ion transmembrane transport (GO:0034220), ion transmembrane transporter activity (GO:0015075), flavonoid metabolic process (GO:0009812), fatty acid binding (GO:0005504), and secondary active sulfate transmembrane transporter activity (GO:0008271). A total of 129 up-regulated candidate genes were annotated into 363 GO pathways, including 234 BP (64.46%), 31 CC (8.54%), and 98 MF (27.00%). The main enrich pathways were transferase activity, transferring hexosyl groups (GO:0016758), oxidoreductase activity, acting on diphenols and related substances as donors (GO:0016679), UDP-glycosyltransferase activity (GO:0008194), cellulose synthase activity (GO:0016759), cellulose synthase (UDP-forming) activity (GO:0016760), and cell wall organization (GO:0071555). In addition, we annotated the DEGs to the KEGG pathway database, and the top 20 pathways with the most significant enrichment were selected and displayed (Supplementary Figure 3B). The main enrich pathways of down-regulated genes were included phenylpropanoid biosynthesis (pper00940), stilbenoid, diarylheptanoid, and gingerol biosynthesis (pper00945), and flavonoid biosynthesis (pper00941); the main enrich pathways of up-regulated genes were included galactose metabolism (pper00052), plant-pathogen interaction (pper04626), glucosinolate biosynthesis (pper00966), linoleic acid metabolism (pper00591), valine, leucine, and isoleucine biosynthesis (pper00290), and pantothenate and CoA biosynthesis (pper00770). Notably, seven down-regulated candidate genes were significantly enriched in phenylpropanoid biosynthesis (pper00940), including two *PpHCT* (PRUPE_3G101000, PRUPE_3G100 800), one *PpCYP98A* (PRUPE_1G580500), one *Pp4CL* (PRU PE_3G107300), one *PpCAOMT* (PRUPE_2G319700), one *PpUDPGT* (PRUPE_8G129800), one *PpPOD* (PRUPE_4G18 6300); in addition, three down-regulated candidate genes were significantly enriched in stilbenoid, diarylheptanoid, and gingerol biosynthesis (pper00945) and flavonoid biosynthesis (pper00941), including two *PpHCT* (PRUPE_3G101000, PRUPE_3G100800) and one *PpCYP98A* (PRUPE_1G580500).

### Analysis of structural genes related to chlorogenic acid biosynthesis

**FIGURE 4:**
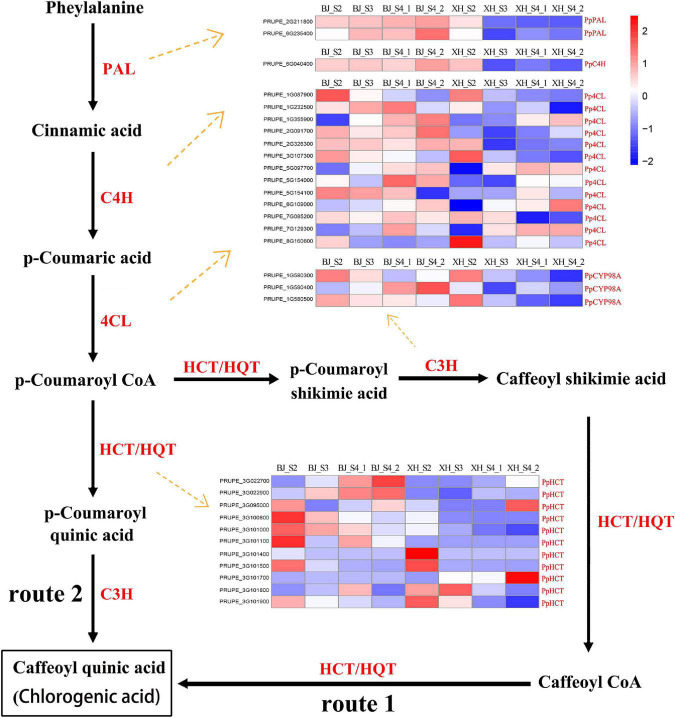
Proposed pathways for the biosynthesis of CGA. The two different routes of CGA biosynthesis were labeled 1 and 2. The names and expression patterns of the enzymes were displayed in each step. PAL, phenylalanine ammonia lyase; C4H, cinnamate 4-hydroxylase; 4CL, 4-coumarate-CoA ligase; C3H, coumarate 3-hydroxylase/cytochrome P450 98A; HCT, hydroxycinnamoyl CoA shikimate/quinate hydroxycinnamoyl transferase; HQT, hydroxycinnamoyl CoA quinate hydroxycinnamoyl transferase. The color scale represented log2 transformed FPKM values. Blue indicated low expression and red indicated high expression.

As reported, there are roughly three pathways in terms of CGA biosynthesis in plants, and these pathways involve many important enzyme-encoding genes. All structural genes involved in CGA synthesis, 2 *PpPAL*, 1 *PpC4H*, 13 *Pp4CL*, 3 *PpCYP98A*, and 11 *PpHCT*, were identified and listed from the transcriptome data (Figure 4 and Supplementary Table 5). Notably, no HCGQT homolog was identified, suggesting that this route for CGA accumulation was most likely to be absent in peach. PAL has been identified as the first enzyme in the phenylpropane metabolic pathway. In this study, we identified two *PpPAL* genes, and their expression in ‘BJ’ exhibited a gradually increasing trend, while that in ‘XH’ showed a decreasing trend. Similarly, *PpC4H* (PRUPE_6G040400) showed an upward trend in ‘BJ’ and a downward trend in ‘XH’. Among 13 *Pp4CL* genes, four *Pp4CL* genes (PRUPE_1G355900, PRUPE_5G097700, PRUPE_6G109000, and PRUPE_7G129300) showed an upward trend in ‘BJ’ and ‘XH’; three *Pp4CL* genes (PRUPE_1G087900, PRUPE_3G107300, and PRUPE_8G160600) showed a decreasing trend in ‘BJ’ and ‘XH’; and one *Pp4CL* gene (PRUPE_2G326300) showed an upward trend in ‘BJ’, while it showed a downward trend in ‘XH’. C3H belongs to the cytochrome P450 monooxygenase superfamily and exists in the form of cytochrome P450 98A2 in peach. Two *PpCYP98A*2 genes (PRUPE_1G580300 and PRUPE_1G580500) showed a downward trend in ‘BJ’ and ‘XH’. In addition, *HCT* and *HQT* are the key genes involved in plant CGA biosynthesis. By comparison with the *HQT* genes in tobacco and tomato, 11 *PpHCT* genes, but no *PpHQT* genes, were obtained from the peach genome. Among them, four *PpHCT* genes (PRUPE_3G100800, PRUPE_3G101000, PRUPE_3G101500, and PRUPE_3G101900) showed a downward trend in ‘BJ’ and ‘XH’, while two (PRUPE_3G027700 and PRUPE_3G027900) showed an upward trend in ‘BJ’ and ‘XH’. In summary, two *Pp4CL* (PRUPE_1G087900 and PRUPE_3G107300), two *PpCYP45098A* (PRUPE_1G580300 and PRUPE_1G580500), and four *PpHCT* genes (PRUPE_3G100800, PRUPE_3G101000, PRUPE_3G101500, and PRUPE_3G101900) presented the same expression trends as the CGAs, indicating that these may be key genes in CGA biosynthesis.

### Screening of transcription factors related to chlorogenic acid biosynthesis

**FIGURE 5:**
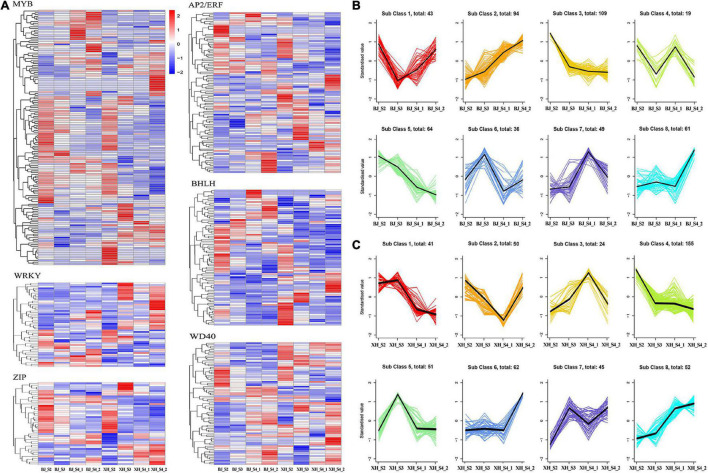
Screening of transcription factors **(A)** gene expression heatmap of 571 transcription factors, **(B)** K-means cluster analysis of transcription factors in ‘BJ’. **(C)** K-means cluster analysis of transcription factors in ‘XH’.

Transcription factors, as transcriptional activators or inhibitors, regulate plant secondary metabolism by binding specifically to cis-acting elements in the promoter region of genes. In this study, we identified 571 TFs, including 172 MYB, 58 WRKY, 120 AP2/ERF, 46 ZIP, 98 bHLH, and 77 WD40 (Figure 5A). K-means cluster analysis indicated nine model expression profiles were obtained in ‘BJ’ and ‘XH’ (Figures 5B,C). In ‘BJ’, Sub Class 3 (109 genes) and Class 5 (64 genes) showed a downward trend, whereas Sub Class 2 (94 genes) showed an upward trend. In ‘XH’, Sub Class 1 (41 genes) and Class 4 (155 genes) showed a downward trend, and Sub Class 8 (52 genes) showed an upward trend. Furthermore, 123 genes showed downward trends in ‘BJ’ and ‘XH’, while 18 genes showed upward trends in ‘BJ’ and ‘XH’ (Supplementary Table 6). Finally, based on the accumulation pattern of CGAs, we identified ten *PpMYB* (PRUPE_1G262700, PRUPE_1G404700, PRUPE_1G405400, PRUPE_2G192100, PRUPE_2G200400, PRUPE_4G062400, PRUPE_4G128000, PRUPE_5G222400, PRUPE_5G045000, and PRUPE_8G164300), one *PpWRKY* (PRUPE_3G308200), one *PpERF* (PRUPE_3G009400), two *PpbHLH* (PRUPE_1G263800 and PRUPE_5G130700), and one *PpWD*40 (PRUPE_6G159500) as candidate TFs for CGA biosynthesis.

### Identification of gene co-expression modules related to chlorogenic acid biosynthesis

**FIGURE 6:**
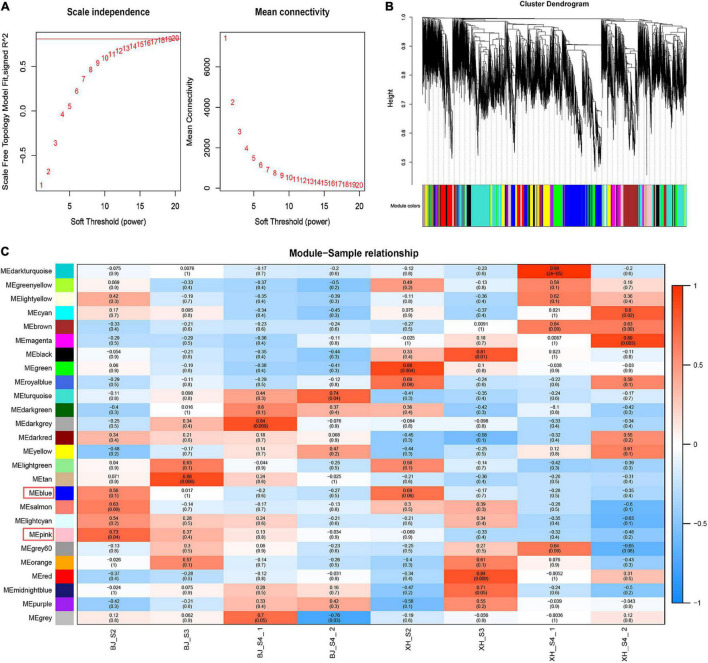
Weighted gene co-expression network analysis. **(A)** Network topology of different soft-thresholding powers. **(B)** Module hierarchical clustering tree. **(C)** Correlation heatmap of samples and modules. Each row corresponded to a module, and each column corresponded to a specific sample. The color of each cell at the row-column intersection indicated the correlation coefficient between the module and sample type, from −1 (blue) to 1 (red).

**FIGURE 7:**
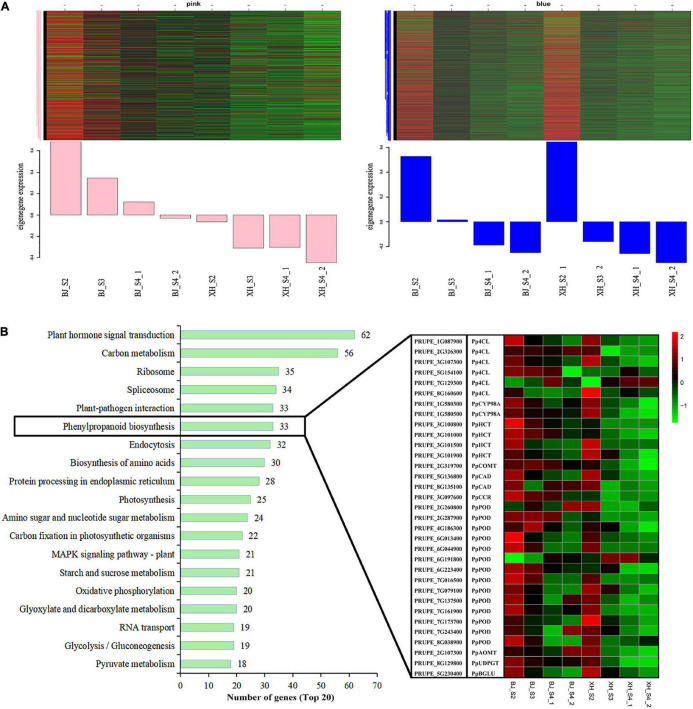
Module expression profiles associated with CGA accumulation. **(A)** Gene expression patterns of the pink and blue modules. Heatmaps showed the expression profile of all co-expressed genes in the module. The *x*-axis indicated the sample type, and the *y*-axis indicated the value of the module eigengene. **(B)** KEGG enrichment of genes in the pink and blue modules. The top 20 enriched pathways were sorted by the number of genes. The boxes represented the expression heatmap of 33 genes of the Phenylpropanoid biosynthesis pathway (pper00940).

Weighted gene co-expression network analysis (WGCNA) was performed using transcriptome data, and a hierarchical clustering tree of 17,654 genes was constructed. Based on the correlation of gene expression with the sample phenotype, a total of 25 co-expression modules (labeled with different colors) were identified, as shown in Figure 6. The largest module (turquoise) was found to contain 3,429 genes, whereas the smallest module (gray) was shown to be comprised of only five genes. Taking the CGA contents as an indicator, the genes highly expressed in ‘BJ’ were found to be concentrated in the pink module, which included 968 genes; and the genes highly expressed in the early stage of fruit development were concentrated in the blue module, which included 3,154 genes (Figure 7A). Furthermore, KEGG pathway enrichment analysis was performed on these 4,122 genes (Supplementary Table 7), and it was found that these genes were significantly enriched in photosynthesis (pper00195), photosynthesis-antenna proteins (pper00196), carbon fixation in photosynthetic organisms (pper00710), and plant hormone signal transduction (pper04075). Notably, 33 genes were enriched in phenylpropanoid biosynthesis (pper00940) (Figure 7B). Among the structural genes of the CGA synthesis pathways, two *PpHCT* (PRUPE_3G100800 and PRUPE_3G101000) were in the pink module, while two *Pp4CL* (PRUPE_3G107300 and PRUPE_1G087900), two *PpCYP45098A* (PRUPE_1G580300 and PRUPE_1G580500), and two *PpHCT* (PRUPE_3G101500 and PRUPE_3G101900) were in the blue module. Among the candidate TFs, one *PpWRKY* (PRUPE_3G308200) and two *PpbHLH* (PRUPE_1G263800 and PRUPE_5G130700) were in the pink modules; while ten *PpMYB* (PRUPE_1G262700, PRUPE_1G404700, PRUPE_1G405400, PRUPE_2G192100, PRUPE_2G200400, PRUPE_4G062400, PRUPE_4G128000, PRUPE_5G222400, PRUPE_5G045000, and PRUPE_8G164300) and one *PpWD40* (PRUPE_6G159500) were in the blue module, which was consistent with our previous screening results.

### Validation of RNA-Seq data by quantitative real-time polymerase chain reaction

To validate the accuracy of the RNA-Seq data, the relative expression levels of the structural genes and TFs related to CGA accumulation, two *PpCYP98A*, three *PpHCT*, and seven *PpMYB*, were examined using qRT-PCR analysis (Supplementary Table 2). For the 12 candidate genes tested, qRT-PCR and RNA-Seq analysis showed similar expression patterns, that is, all genes showed a declining expression trend, and the expression level of genes in ‘BJ’ was higher than that in ‘XH’ (Supplementary Figure 4). All in all, these results indicated that the RNA-Seq data could represent the relative expression levels of genes at different developmental stages in peach fruits, and the candidate genes and TFs related to CGA accumulation identified in this study were reliable.

## Discussion

### Identification and evaluation of chlorogenic acids in peach fruit

As the main phenolic compounds, CGAs exist in different isomeric forms, including monoCQAs (3-caffeoylquinic acid, 4-caffeoylquinic acid, and 5-caffeoylquinic acid), diCQAs (1,3-dicaffeoylquinic acid, 3,4-dicaffeoylquinic acid, 3,5-dicaffeoylquinic acid, and 4,5-dicaffeoylquinic acid), triCQAs (3,4,5-tricaffeoylquinic acid), methyl-3-O-caffeoylquinic acid, and ethyl-3-O-caffeoylquinic acid (29). Progress has been made in identifying the component and content identification of CGAs using high performance liquid chromatography/high performance liquid chromatography-mass spectrometry, and numerous studies have shown that CGA is the most abundant phenolic acid in various fruit, such as peach, blueberry, pear, strawberry, and apple (5–8). In this study, we assessed the content and composition of CGAs in the mature fruits of 205 peach cultivars. Three forms of CGAs were identified: CGA (52.57%), NCGA (47.13%), and CCGA (0.30%). Among these, CCGA was only reported in blood-flesh peaches (30).

There were significant differences in the contents of CGAs in different flesh-color of peaches. The contents of CGAs in blood flesh peaches were significantly higher than that of white and yellow flesh peaches, which was consistent with the results of previous studies (25). We concluded that blood flesh peach not only contained high anthocyanins, but also contained high phenolic acids, so it had higher antioxidant capacity, and could be used as specific germplasms for extracting active substances. The contents of CGAs in different fruit types of peaches were also different. Lu et al. determined the phenolic compounds of 15 peach cultivars and found that epicatechin and CGA were the main phenolic compounds of honey peach; epicatechin, catechin, CGA, and NCGA were the main phenolic compounds of flat peach; CGA and catechin were the main phenolic compounds of nectarine (31), which was somewhat different from the results of our studies. We speculated that this might be due to differences in the genetic background of the selected peach cultivars, which also suggested that the number of accessions should be expanded in the future evaluation of germplasm resources. Moreover, Cai et al. found that the phenolic contents of different pulp textures were different, the phenolic contents of crisp peach were significantly higher than in melting and stony hard peach (26). Related research has shown that the crisp peach evolved from wild peach, and most of the wild types have the characteristics of bitter taste and high phenolic content (32).

In addition, it was found that the contents of CGAs in peel and flesh were significantly different. The contents of total CGAs in the peel of peach were significantly higher than those in the flesh. Similarly, the CGA of apple fruit was mainly present in the core area and the seeds with an intermediate level in the flesh and a low level in the skin (8), which indicated that the synthesis and accumulation of CGAs were tissue-specific in different organs. Overall, these results indicated the diversity of CGAs in different peach germplasm resources, which will be beneficial for screening special germplasm resources and will provide a reference for germplasm resource evaluation and quality breeding in peach.

### Dynamic patterns of metabolites involved in the chlorogenic acid synthesis pathways in peach fruit

The phenylpropane metabolic pathway is important to the synthesis of secondary metabolites in plants, and it is associated mainly with the synthesis of downstream secondary metabolites, such as CGA, lignin, and anthocyanin (29). To investigate the rules of accumulation for various metabolites involved in the CGA synthesis pathways during peach fruit development, two high-CGAs cultivars (‘BJ’ and ‘HYT’) and two low-CGAs cultivars (‘XH’ and ‘RG’) were selected. It was worth noting that the trends in the accumulation of CGAs in the four peach fruit cultivars were relatively consistent. With the growth and development of peach fruits, the content of total CGAs generally showed a trend toward rising first and then decreasing. The content of CGAs in the four peach fruit varieties was the highest at the young fruit stage (S1) and gradually decreased at the later fruit development stage (S2–S4). The same conclusions have been verified in tomato, pear, and apple (5, 8). However, due to the different growth stages of the four peach cultivars, the peaks in the accumulation of the total CGAs differed. Therefore, we hypothesized that CGAs were synthesized and accumulated during the early stage of fruit development and degraded during the later stage of fruit development. As important participating metabolomes and intermediate metabolites of the CGA synthesis pathway, quinic acid, shikimic acid, p-coumaroyl quinic acid, and caffeoyl shikimic acid all showed similar dynamic patterns to that of the CGAs, although their accumulation peaks occurred slightly earlier; moreover, they were all highly expressed in ‘BJ’ and ‘HYT’ but expressed at low levels in ‘XH’ and ‘RG’. Thus, we presumed that these metabolomes might provide the precursor material basis for the accumulation of CGAs in the later stages of fruit development.

Previous reports have noted that the biosynthetic pathways of Lignin and CGA shared intermediates and enzymes, and one of the major routes to CGA synthesis occurs *via* the shikimate shunt, which is also the major route toward the synthesis of G and S lignin units (33, 34). CGA was frequently regarded as authentic intermediates of the lignin biosynthetic pathway, but this view remains controversial. During the early development of *Capsicum annuum* seedlings, a decrease in CGA content in hypocotyls is accompanied by a significant increase in lignin deposition (35). In switchgrass stems, the content of CGA and lignin also showed opposite accumulation patterns, young internodes show lower lignin content compared to mature internodes, whereas the opposite is found for the CGA content (36). These researches seem to indicate that there is some competition between CGA and lignin. However, the metabolic pathways involved in CGA and lignin synthesis appear to be fully independent in *Nicotiana tabacum*. *NtHQT* gene silencing produced relatively normal plant phenotypes, with CGA levels reduced and no effect on lignin, While *NtHCT* gene silencing produced markedly altered phenotypes, with lignin levels partially reduced and no effect on CGA (37). In our study, lignin showed similar dynamic changes in accumulation to CGA during the growth and development of peach fruit, although its peak in accumulation was later than that of the CGA. There is a certain positive correlation between lignin and CGA during the growth and development of peach fruit. However, the lignin contents showed no correlations among different peach varieties. Therefore, we guessed that there was a certain relationship between CGA and lignin, but it was not dependent.

Another report indicated that the texture differences in peach fruit might be the result of differential expression of two branches of the phenylpropanoid metabolic pathway. One branch, which regulates flavonoid metabolism, was highly active in ‘Hongli’ fruit; whereas the other branch, which regulates lignin synthesis, was more highly active in ‘Baili’ fruit (38). However, we came to different conclusions in this study. Only in ‘BJ’ (dominant blood-flesh, DBF), ‘XH’ (white-flesh), and ‘RG’ (yellow-flesh), did anthocyanin show an opposite trend to CGA and lignin accumulation, which seemed to be the result of the differential expression of two branches of the phenylpropanoid metabolic pathway. However, the trend of anthocyanin was the same as that for CGA and lignin in ‘HYT’ (blood-flesh, bf). Moreover, peaches with a high anthocyanin content exhibited a higher content of CGA, indicating that CGAs and anthocyanin might have a certain positive correlation. In conclusion, the regulation of CGAs, lignin, and anthocyanins is complex, and these compounds work together to maintain a dynamic balance.

### Key genes for chlorogenic acid synthesis in peach fruit

Chlorogenic acid metabolism is controlled mainly by two types of genes: structural genes and regulatory genes. Using high-throughput sequencing, we identified 30 structural genes involved in the first and second pathways of CGA biosynthesis, including 2 *PpPAL*, 1 *PpC4H*, 13 *Pp4CL*, 3 *PpCYP98A*, and 11 *PpHCT*. As in most other plant species, no HCGQT (UGCT) homologous gene was identified, indicating that the CGA synthesis route of caffeoyl glucoside and quinic acid may not exist in peach.

*PAL* and *C4H* are key genes of the upstream phenylpropane metabolic pathway, they are not only involved in CGA biosynthesis but also participate in the biosynthesis of precursor compounds that catalyze other metabolic pathways, such as those for anthocyanin, salvianolic acid, flavone, and flavonol biosynthesis (39). In this study, two *PpPAL* and one *PpC4H* showed a downward trend in ‘XH’ (*white* flesh peach) and a gradual upward trend in ‘BJ’ (red flesh peach). Therefore, we speculated that *PpPAL* and *PpC4H* might be involved in other metabolic pathways (such as anthocyanin biosynthesis) and not be the key gene for CGA biosynthesis in peach. In addition, we found that there is some functional redundancy between different members of the same gene family. The expression levels of only two *Pp4CL*, two *PpCYP98A*, and four *PpHCT* genes were positively correlated with the content of CGA, indicating that these may be key genes for CGA biosynthesis. However, the expression levels of other genes (11 *Pp4CL*, 1 *PpCYP98A*, and 7 *PpHCT*) showed no correlation with the content of CGA or were extremely low, indicating that these genes had functional redundancy and were not key genes regulating CGA biosynthesis.

It was worth noting that both HCT and HQT belong to the BAHD acetyltransferase superfamily, with acyl receptor specificity, but due to their different enzymology and molecular expression characteristics, their roles and functions in different plants differ (40). HCT can use shikimic acid and quinic acid as acyl acceptors simultaneously, but HQT has a significant preference for quinic acid and HCT for shikimic acid (41). HCT is a key enzyme in the biosynthesis pathway of phenylpropane, and it is involved in the biosynthesis of lignin. The down-regulation of the HCT gene in *A. thaliana* and *Medicago sativa* leads to a significant decrease in the level of lignin (42). In addition, HCT participates in CGA biosynthesis in artichoke, honeysuckle, and pear (3, 5). HQT is only involved in the biosynthesis of CGA, and its biological functions have been verified in many plants such as tobacco, tomato, artichoke, potato, and honeysuckle (2). By comparing with HQT homologous genes in tobacco and tomato, 11 *PpHCT* genes were found in the peach genome, but no HQT genes. At the same time, we found that, as the acyl receptor substrate of HCT, the content of quinic acid in peach was significantly higher than that of shikimic acid. Therefore, we speculate that the HCT enzyme properties of peach may be similar to the HQT enzyme properties of tobacco and tomato, that is, HCT has a higher affinity for quinic acid, but its specific enzymatic characteristics and biological functions need to be further verified.

Transcription factors, such as MYB, WRKY, and ERF, can activate or inhibit the expression of structural genes, thereby regulating CGA biosynthesis (17, 21, 22). In this study, we identified 571 TFs, including 172 MYB, 58 WRKY, 120 AP2/ERF, 46 ZIP, 98 bHLH, and 77 WD40. Through K-means cluster analysis, 15 TFs (10 *PpMYB*, 1 *PpWRKY*, 1 *PpERF*, 2 *PpbHLH*, and 1 *PpWD40*) were initially screened. Furthermore, two gene co-expression modules related to CGA biosynthesis were identified by WGCNA. Among the candidate TFs, three were in the pink modules, while 11 were in the blue module. Interestingly, we screened many MYB TFs, whose biological functions in regulating CGA biosynthesis have been validated in many plants. In suspension-cultured carrot cells, *DcMYB3* and *DcMYB5* activated the transcription of *DcPAL3*, resulting in a high level of CGA accumulation (19). In tomato, *AtMYB11* and *AtMYB12* regulate the biosynthesis of caffeoyl quinic acid and flavonol (43). In *Lonicera macranthoides*, *LmMYB15* activates the promoters of *4CL*, *MYB3*, and *MYB4*, thereby facilitating CGA biosynthesis and phenylpropanoid metabolism (44). However, the regulatory mechanism has not been reported in peach, and further functional validation of candidate genes and regulatory elements is required to elucidate a complete picture.

## Conclusion

In this study, we evaluated the composition and content of CGAs in mature fruits of 205 peach cultivars and the accumulation patterns of metabolites in the CGA biosynthesis pathways throughout the fruit development of four cultivars. In peach fruits, three forms of CGA (52.57%), NCGA (47.13%), and CCGA (0.30%) were identified. The contents of CGAs in different flesh colors and fruit types of peach germplasm resources were diverse. During the growth and development of peach fruits, the content of CGAs generally showed a trend of rising first and then decreasing. Notably, quinic acid, shikimic acid, p-coumaroyl quinic acid, and caffeoyl shikimic acid all showed similar dynamic patterns to CGA, which might provide the precursor material basis for the accumulation of CGA in the later stage. Moreover, CGA, lignin, and anthocyanins might have a specific correlation and these compounds work together to maintain a dynamic balance. By the comparative transcriptome analysis, eight structural genes (*Pp4CL*, *PpCYP98A*, and *PpHCT*) were positively correlated with the content of CGA, which were involved in the first and second routes of CGAs biosynthesis. In addition, 15 regulatory genes (*PpMYB*, *PpWRKY*, *PpERF*, *PpbHLH*, and *PpWD40*) were also initially screened as candidate TFs for CGAs biosynthesis. Our findings provide new insights into the metabolic and regulatory mechanisms of CGA biosynthesis in peach fruit and will inform future efforts to develop high CGA content peaches in future breeding programs.

## Data availability statement

The original contributions presented in this study are included in the article/Supplementary Material, further inquiries can be directed to the corresponding author. The data presented in this study are deposited in the NCBI SRA repository, accession number PRJNA850935 (https://submit.ncbi.nlm.nih.gov/subs/sra/SUB11621949/overview).

## Author contributions

ZSu and JY conceived and designed the research and drafted the manuscript. HJ, MS, and ZC performed the experiments. BZ and JL analyzed the data. ZSh, RM, and MY revised the manuscript. All authors have read and approved the final manuscript.

## Conflict of interest

The authors declare that the research was conducted in the absence of any commercial or financial relationships that could be construed as a potential conflict of interest.

## Publisher’s note

All claims expressed in this article are solely those of the authors and do not necessarily represent those of their affiliated organizations, or those of the publisher, the editors and the reviewers. Any product that may be evaluated in this article, or claim that may be made by its manufacturer, is not guaranteed or endorsed by the publisher.

## Footnotes

1
http://geneontology.org/

2
https://www.kegg.jp/

## Supplementary material

Supplementary Figure 1 HPLC chromatogram of chlorogenic acids standard sample (100 mg/kg).

Supplementary Figure 2 Comparison of CGAs in different peach germplasm resources. **(A)** Comparison of CGAs in different flesh-color peaches. **(B)** Comparison of CGAs in different fruit types peaches.

Supplementary Figure 3 Enrichment analysis of the DEGs. **(A)** GO enrichment analysis. **(B)** KEGG pathway enrichment analysis.

Supplementary Figure 4 The validation of RNA-Seq data of 12 candidate genes by qRT-PCR.
