# Supplementary figures and images for: Integrative analysis of the metabolome and transcriptome reveals the molecular mechanism of chlorogenic acid synthesis in peach fruit
Source: Front Nutr. 2022 Jul 19;9:961626. doi: 10.3389/fnut.2022.961626 (PMC9344011; doi:10.3389/fnut.2022.961626)

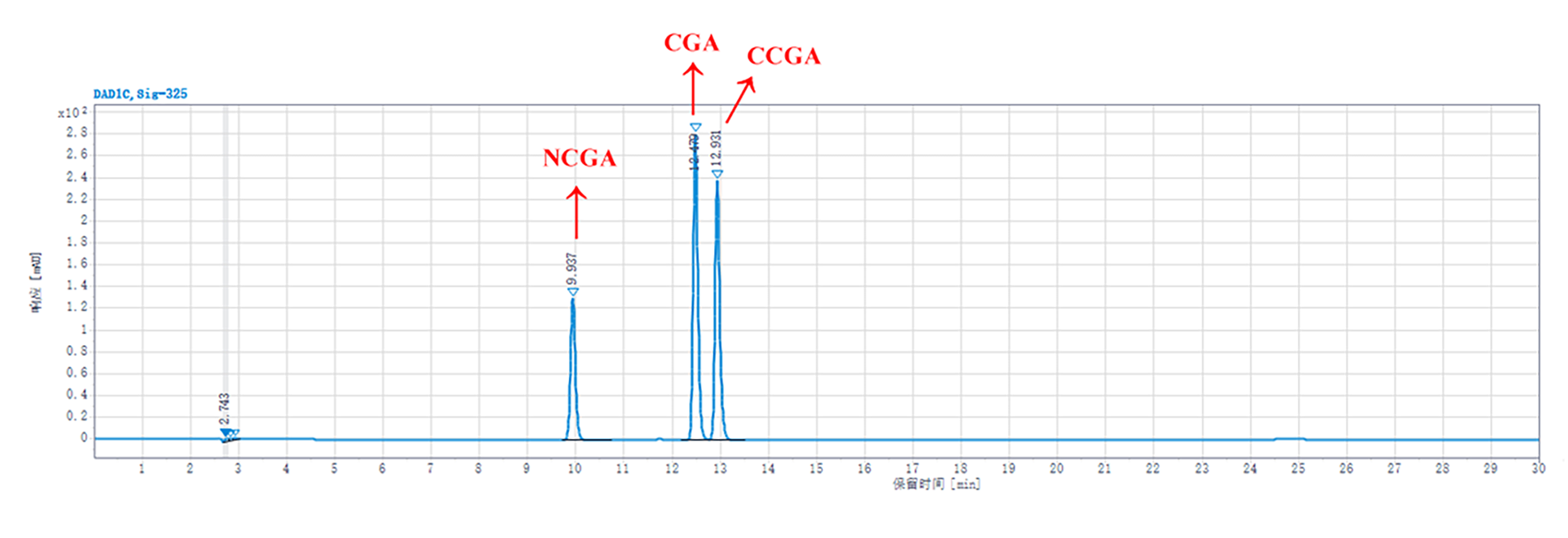

Supplement: Supplementary Figure 1 — HPLC chromatogram of chlorogenic acids standard sample (100 mg/kg). [file Image_1.tif]

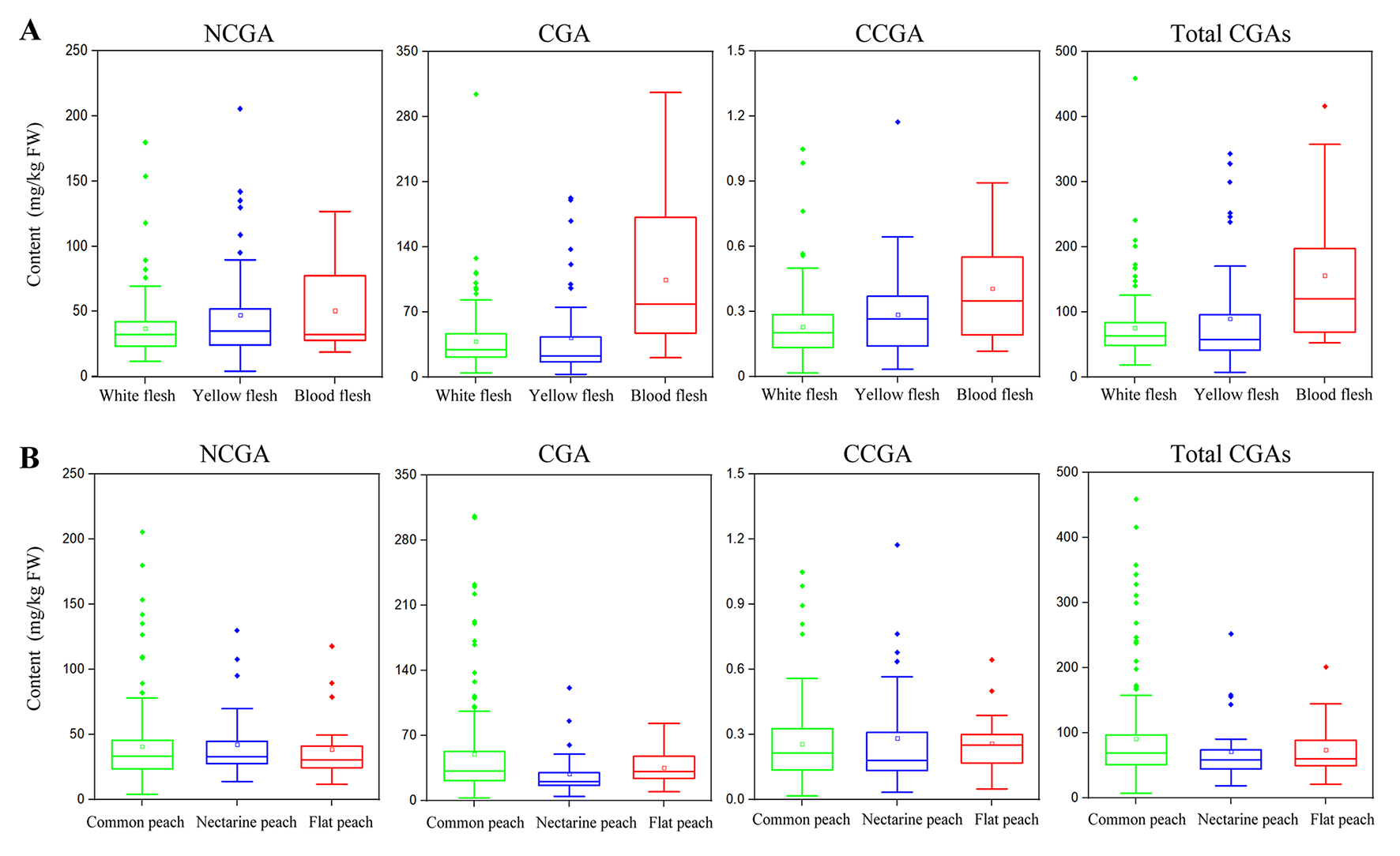

Supplement: Supplementary Figure 2 — Comparison of CGAs in different peach germplasm resources. (A) Comparison of CGAs in different flesh-color peaches. (B) Comparison of CGAs in different fruit types peaches. [file Image_2.tif]

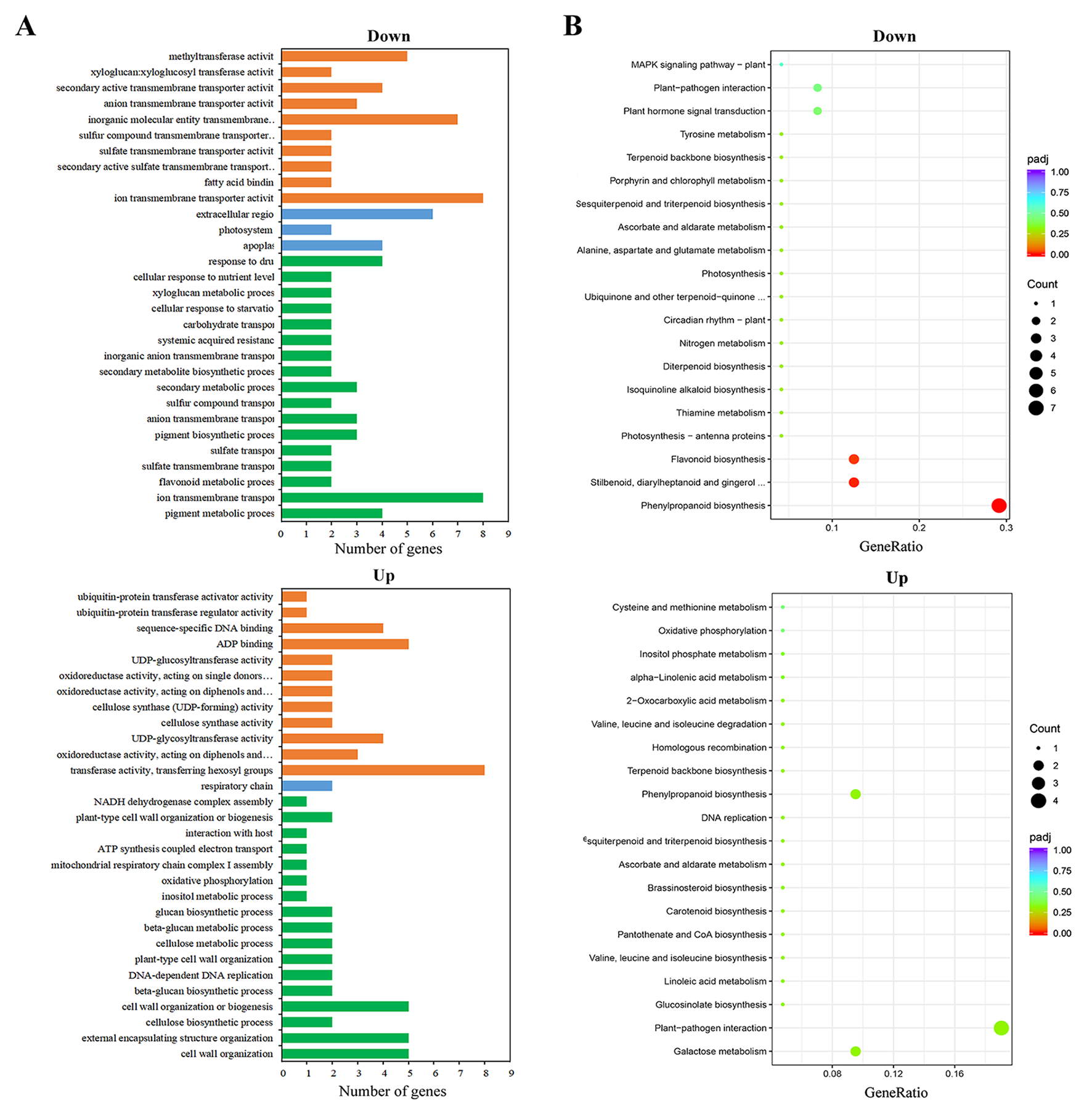

Supplement: Supplementary Figure 3 — Enrichment analysis of the DEGs. (A) GO enrichment analysis. (B) KEGG pathway enrichment analysis. [file Image_3.tif]

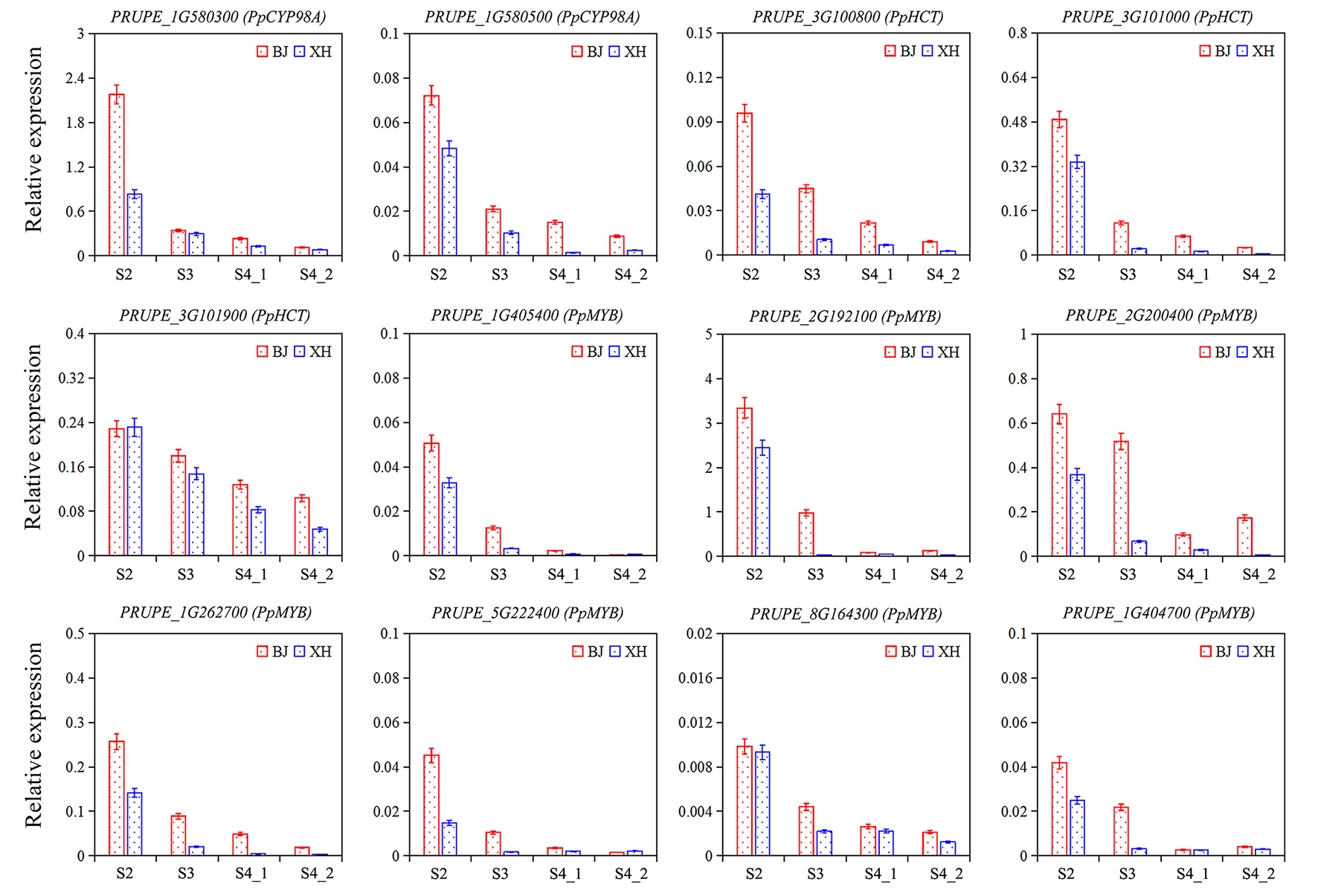

Supplement: Supplementary Figure 4 — The validation of RNA-Seq data of 12 candidate genes by qRT-PCR. [file Image_4.tif]
